# A Novel 3D Node Deployment Inspired by Dusty Plasma Crystallization in UAV-Assisted Wireless Sensor Network Applications

**DOI:** 10.3390/s21227576

**Published:** 2021-11-15

**Authors:** Rongxin Tang, Yuhao Tao, Jiahao Li, Zhiming Hu, Kai Yuan, Zhiping Wu, Shiyun Liu, Yuhao Wang

**Affiliations:** 1 Institute of Space Science and Technology, Nanchang University, Nanchang 330031, China; rongxint@ncu.edu.cn (R.T.); taoyuhao17@gmail.com (Y.T.); 2 School of Information and Engineering, Nanchang University, Nanchang 330031, China; 6002119021@email.ncu.edu.cn (J.L.); wangyuhao@ncu.edu.cn (Y.W.); 3 Jiangxi Provincial Key Laboratory of Interdisciplinary Science, Nanchang University, Nanchang 330031, China; 4 CAICT (Jiangxi) Science and Technology Innovation Research Institute Co., Ltd., Nanchang 330031, China; huzhiming@caict.ac.cn; 5 Computing Institute of Jiangxi Province, Nanchang 330031, China; wuzp218@163.com; 6 Usun Microelectronics, Nanchang 330031, China; mackliu@usunmicro.com

**Keywords:** 3D node deployment, wireless sensor networks, dusty plasma crystallization

## Abstract

With the rapid progress of hardware and software, a wireless sensor network has been widely used in many applications in various fields. However, most discussions for the WSN node deployment mainly concentrated on the two-dimensional plane. In such a case, some large scale applications, such as information detection in deep space or deep sea, will require a good three dimensional (3D) sensor deployment scenario and also attract most scientists’ interests. Excellent deployment algorithms enable sensors to be quickly deployed in designated areas with the help of unmanned aerial vehicles (UAVs). In this paper, for the first time, we present a three dimensional network deployment algorithm inspired by physical dusty plasma crystallization theory in large-scale WSN applications. Four kinds of performance evaluation methods in 3D space, such as the moving distance, the spatial distribution diversion, system coverage rate, and the system utilization are introduced and have been carefully tested.Furthermore, in order to improve the performance of the final deployment, we integrated the system coverage rate and the system utilization to analyze the parameter effects of the Debye length and the node sensing radius. This criterion attempts to find the optimal sensing radius with a fixed Debye length to maximize the sensing range of the sensor network while reducing the system redundancy. The results suggest that our 3D algorithm can quickly complete an overall 3D network deployment and then dynamically adjust parameters to achieve a better distribution. In practical applications, engineers may choose appropriate parameters based on the sensor’s hardware capabilities to achieve a better 3D sensor network deployment. It may be significantly used in some large-scale 3D WSN applications in the near future.

## 1. Introduction

Wireless sensor network (WSN) has been a hot research topic in the recent decades, as it provides a fundamental communication tunnel and information collection from different environments [1]. WSNs have been widely used in daily life or industry area, such as the detection of temperature, pressure, traffic, power grid, and a patient’s physical signs. Many WSN application scenarios have entered the mature stage and various application modes have been emerging, one after another [2,3,4]. For example, the information in a smart grid can be provided to power companies through a wireless sensor system and achieve high system efficiency and a feasible scheme for state monitoring [5,6]. Losilla et al. tried to reduce the cost of intelligent transportation system and enhance its intelligence by using WSNs [7]. Rashid et al. discussed a series of WSN applications in urban areas [8]. Aqeel et al. created and applied one scenario of WSN in the field of agriculture [9]. Obviously, the rapid development of unmanned applications make the WSN become an indispensable issue in the near future.

Usually, engineers have to design the best scenario for the sensor locations. The most essential aspect in designs and applications of the WSNs is the large scale node deployment. Virtual force algorithm (VFA) is a novel algorithm strategy for sensor deployment using virtual force, which is pursued by many scientists. Wang et al. proposed a dynamic deployment strategy, which can take both the historical local information and virtual forces for sensor into consideration [10]. In order to increase the converge speed, Chen et al. added an expression of exponential function for the relationship of virtual force [11]. Yu et al. introduced a strategy that, only if two nodes in the Delaunay graph are connected, the neighboring relationship of WSN nodes can be well defined and the virtual force can only be applied from neighboring nodes within the communication range [12]. By introducing the force model of centripetal force and grid theory, Zhang et al. proposed a coverage enhancement algorithm based on a virtual centripetal force, which can effectively turn off the redundant sensors and improves the coverage effect [13].

However, most of the recent WSN node deployment strategies are mainly for a two dimensional plane. In real applications, an effective three dimensional algorithm for large scale network deployment still requires further attention. Recently, Tan et al. introduced a 3D spatial self-deployment algorithm which uses a weighted Voronoi diagram (3dv-hdda) to move the sensor nodes and introduces a virtual boundary torque to rotate sensor nodes to reach the best positions [14]. Du et al. proposed a heuristic algorithm suitable for limited communication environment [15]. The selected redundant sensor nodes can move to their selected areas to improve the efficiency of WSN coverage and connectivity. For different *k* coverage requirements to achieve uneven regional coverage optimization, Wang et al. proposed a *k*-equivalent radius enhanced virtual force algorithm (called k-ervfa) [16]. Miao et al. introduced a negotiation strategy to ensure the network connectivity and also used a density control strategy to balance all the distributed sensor nodes [17]. Referring to an artificial potential-field theory, Lv et al. proposed a three-dimensional dynamic path planning algorithm for mobile anchor nodes based on virtual force [18]. This algorithm indicates that the movements of anchor nodes by calculating the virtual force can be exerted by unknown anchor nodes in different regions. Interestingly, based on the phenomenon that dust particles can automatically form Yukawa crystal structure, Tang et al. presented an adaptive deployment algorithm for large-scale WSN node deployment and discussed in detail the effects of calculation scale and shielding length on the algorithm [19,20]. This algorithm performs well and has good spatial expansibility, which can be expanded to a 3D scenario.

The unmanned aerial vehicle (UAV) industry has been growing at a tremendous speed in the recent years. Sensor nodes can be carried on the UAVs to inaccessible locations and be used in a lot of real applications, such as desert, deep sea, or deep space, which can substantially expand people’s ability to detect information. All those applications may require a 3D node deployment for a relatively large-scale sensor network [21]. Based on the UAV’s high mobility, Li et al. [22] designed an equal service distance algorithm to maintain a superior surveillance performance. Ryu et al. [23] proposed a system inspired from the biological cell differentiation through hormones that can coordinate the sensing effort to form a distributed sensor network for UAVs. Elmokadem [24] introduced a distributed coverage control laws to guarantee optimal sensor self-deployment over three-dimensional sensing fields. However, the UAV-assisted 3D WSN still lacks good node deployment algorithms for its further applications.

It is well known that the optimal structure of a sensor network in a two-dimensional plane is a regular hexagonal network structure, but the optimal coverage model in three-dimensional space is still controversial. Bambah and Gupta already demonstrated that the node utilization of the body-centered cube structure (BCCS) is up to 68.3%, which indicates that the spherical coverage model of the body-centered cube structure may become the most economical one in the three-dimensional space [25]. The Voronoi units of all nodes in the spherical coverage model of the body-centered cube are the truncated octahedrons, which can also be infinitely stitched into a larger three-dimensional space and form the most thermodynamically stable crystal. Jiang et al. have truncated the octahedron vertices to locate sensor nodes to achieve a 3D WSN with the fewest nodes [26]. This deployment algorithm will obtain seamless sampling coverage and high connectivity at the same time. Felamban et al. filled a 3D space with a truncated octahedron for target-range coverage to improve the performance limitations in underwater communications via sound waves [27]. Experimental results by Alam et al. [28] and Xiang et al. [29] suggest that, for a seamless 3D structure with minimal number of nodes, a truncated octahedron is the best choice for filling the defined space.

Therefore, the spherical model with truncated octahedrons as structure units in space is a good 3D coverage model. In this paper, for the first time, we present a three dimensional network deployment algorithm inspired by physical dusty plasma crystallization theory in large-scale WSN applications. The 3D node deployment algorithm (VFA-DP-3D) and corresponding equations developed from the dusty plasma theory are presented. We also introduced four kinds of evaluation methods in 3D space, such as the moving distance, the spatial distribution diversion, the system coverage rate and the system utilization. We used different ways to analyze the final performance of our 3D WSN deployment based on these four conditions. These evaluation methods can importantly present the validation of the 3D WSN deployment. It can be clearly seen from all experiments that our VFA-DP-3D algorithm can successfully achieve a better spherical stack of truncated octahedrons in a 3D space, which may have a maximum coverage rate of 0.8. Furthermore, the parameter effects of the Debye length and the node sensing radius have also been discussed. We innovatively combined the system coverage rate and the system utilization to find an optimal sensor radius and improve the final deployment network if the sensor network has the largest perception range and relatively small system redundancy. Our algorithm gives new insights into the 3D WSN deployment based on a physical law, e.g., dusty plasma crystallization, which may be combined with other self-deployment methods to improve their location accuracy and achieve a hybrid optimization.

The rest of this paper was organized as follows. The related 3D algorithm are introduced in Section 2. Section 3 proposes the methodology of performance evaluation. The simulation experiments and different parameter effects during the deploying process are presented in Section 4 and Section 5. Finally, the conclusions are summarized in Section 6.

## 2. Virtual Force Algorithm Based on Plasma Yukawa Potential Model

Generally, one sensor node should include both the sensor (for collecting and exchanging information) and the movement devices (such as the GPS equipped by the UAV itself). In our paper, we consider all these hardware devices as a whole sensor node. The dust plasma can generally form stable regular hexagonal lattices in 2D plane in the physical world, which can be used for the the optimal coverage model of the wireless sensor deployment in a virtual force algorithm. Here we mainly concentrated on a theoretical algorithm, which presents a possible 3D node deployment strategy in simulations. Due to the self-crystallizing feature of dust plasma under the effect of plasma Yukawa potential, we exploit this capability to deploy sensor nodes in an innovative way. Each sensor carried by an unmanned aerial vehicle is regarded as a dust particle in our VFA-DP-3D deployment algorithm. The node position information will then be computed by the virtual force (in the dusty plasma system) of the surrounding nodes.

Based on the molecular dynamics simulation, when a sensor node is regarded as a dust particle, the corresponding motion equation can be written as follows:

(1) $$m\frac{d^{2}{\vec{r}}_{i}}{dt^{2}}=-\nabla \phi_{i}-m\gamma \frac{d{\vec{r}}_{i}}{dt}$$

or

(2) $$m{\vec{a}}_{i}=-\nabla \phi_{i}-m\gamma {\vec{v}}_{i},$$

where *m* is mass of the particle, ${\vec{r}}_{i}$ is the displacement vector of particle *i* and γ is the friction coefficient. It should be noted that the gradient $\nabla \phi_{i}$ of the electrostatic potential $\phi_{i}$ in 3D Cartesian Coordinates can be expressed as $\nabla \phi_{i}=\left(\partial \phi_{i}/\partial x,\partial \phi_{i}/\partial y,\partial \phi_{i}/\partial z\right)$. Therefore, the formula of $\phi_{i}$ in a 3D space is

(3) $$\begin{array}{ccc} \phi_{i} & = & k{\vec{r}}_{i}^{2}\left(t\right)+\sum_{i\neq j}^{N}\frac{Q^{2}}{4\pi \varepsilon_{0}r_{ij}}e^{-\frac{r_{ij}}{\lambda_{D}}}\\ & & =k\left(x_{i}^{2}+y_{i}^{2}+z_{i}^{2}\right)+\sum_{i\neq j}^{N}\frac{Q^{2}}{4\pi \varepsilon_{0}}\\ & & \;\cdot {\left[{\left(x_{i}-x_{j}\right)}^{2}+{\left(y_{i}-y_{j}\right)}^{2}+{\left(z_{i}-z_{j}\right)}^{2}\right]}^{-1/2}\\ & & \;\cdot e^{-\frac{{\left[{\left(x_{i}-x_{j}\right)}^{2}+{\left(y_{i}-y_{j}\right)}^{2}+{\left(z_{i}-z_{j}\right)}^{2}\right]}^{1/2}}{\lambda_{D}}}. \end{array}$$

where *k* is the spring coefficient, $r_{i}$ is the radial distance of particle *i*, *Q* is the amount of charge, $r_{ij}$ is the distance from particle *i* and particle *j*, $\lambda_{D}$ is the Debye length of the plasma and *N* is the total number of all particles in the communication range. On the right side of Equation (3), the first term $k{\vec{r}}_{i}^{2}$ is the external harmonic potential, which provides the radial constraint so that the spring constant *k* can regulate the separation between particles; the second term $\left(Q^{2}/\left(4\pi \varepsilon_{0}r_{ij}\right)\right)\exp\left(-r_{ij}/\lambda_{D}\right)$ is a Yukawa potential. When $r_{ij}$ is greater than $\lambda_{D}$, the Yukawa potential quickly decreases and the interactions between the particles become very weak. This means that $\lambda_{D}$ is the shielding length which controls the range of particle interactions in the system. Σ is the sum of the Yukawa potential of particle *i* subjected to other particles in the computational scale $R_{c}$. In actual WSN applications, $\lambda_{D}$ is usually chosen to be far less than the communication distance $R_{c}$ so that the interaction between two nodes is manly obtained from the effect of Yukawa potential.

In order to analyze how all nodes in wireless sensor networks are deployed from the initial random distribution to the final steady topology, the molecular dynamic equations are used for simulations in this paper. A second-order leap-frog scheme was adopted and the time discretization operation is divided into the time step dt. The force on each node is constant in dt and results in a uniform motion. The formulae are as follows

(4) $${\vec{r}}_{n+1}={\vec{r}}_{n}+{\vec{v}}_{n}dt+{\vec{a}}_{n}\frac{dt^{2}}{2}$$

(5) $${\vec{v}}_{n+1}={\vec{v}}_{n}+\left({\vec{a}}_{n}+{\vec{a}}_{n+1}\right)\frac{dt}{2}$$

where *r*, *v* and *a* are the node position, velocity, and acceleration at each time step, respectively. Substituting Equations (2) into (4) and (5), we can obtain

(6) $${\vec{r}}_{n+1}={\vec{r}}_{n}+{\vec{v}}_{n}dt+\left(-\frac{\nabla \phi_{i}}{m}-\gamma {\vec{v}}_{n}\right)\frac{dt^{2}}{2},$$

(7) $${\vec{v}}_{n+1}={\vec{v}}_{n}+\left(-\frac{2\nabla \phi_{i}}{m}-\gamma {\vec{v}}_{n}-\gamma {\vec{v}}_{n+1}\right)\frac{dt}{2}.$$

Since the three-dimensional representation of the gradient ∇ can be expressed by $\nabla =\left[\partial /\partial x\right]\vec{i}+\left[\partial /\partial y\right]\vec{j}+\left[\partial /\partial z\right]\vec{k}$ in Cartesian coordinate, the expression of Equation (2) can be written as $ma_{x}=-\partial \phi_{i}/\partial x_{i}-m\gamma v_{x}$ in *x*-axis. According to Equation (3), we have

(8) $$\begin{array}{ccc} \frac{\partial \phi_{i}}{\partial x_{i}} & = & 2kx_{i}+\sum_{i\neq j}^{N}\left[\frac{-Q^{2}}{4\pi \varepsilon_{0}r_{ij}^{2}}\frac{x_{ij}}{r_{ij}}e^{-\frac{r_{ij}}{\lambda_{D}}}+\frac{-Q^{2}}{4\pi \varepsilon_{0}r_{ij}^{2}}\frac{x_{ij}}{\lambda_{D}}e^{-\frac{r_{ij}}{\lambda_{D}}}\right]\\ & = & 2kx_{i}+\sum_{i\neq j}^{N}\frac{Q^{2}}{4\pi \varepsilon_{0}}\left(\frac{1}{r_{ij}}+\frac{1}{\lambda_{D}}\right)\left(-\frac{x_{ij}}{r_{ij}^{2}}\right)e^{-\frac{r_{ij}}{\lambda_{D}}} \end{array}$$

and

(9) $$ma_{x}=-2kx_{i}-m\gamma v_{x}-\sum_{i\neq j}^{N}\frac{Q^{2}}{4\pi \varepsilon_{0}}\left(\frac{1}{r_{ij}}+\frac{1}{\lambda_{D}}\right)\left(-\frac{x_{ij}}{r_{ij}^{2}}\right)e^{-\frac{r_{ij}}{\lambda_{D}}}.$$

By the same operation as above for Equations (6) and (7), we can also obtain

(10) $$r_{(+1)x}=r_{x}+v_{x}dt-\frac{1}{2}\left(\frac{1}{m}\frac{\partial \phi_{i}}{\partial x_{i}}+\gamma v_{x}\right)dt^{2}$$

(11) $$v_{(+1)x}=v_{x}-\frac{1}{2}\left(\frac{2}{m}\frac{\partial \phi_{i}}{\partial x_{i}}+\gamma v_{x}+\gamma v_{(+1)x}\right)dt.$$

Substituting (8) into (11) we then obtain the velocity for the next time step

(12) $$\begin{array}{c} \left(1+\frac{\gamma dt}{2}\right)v_{(+1)x}=v_{x}-\left(\frac{\gamma }{2}v_{x}+\frac{2k}{m}x_{i}\right)dt+\sum_{i\neq j}^{N}\frac{Q^{2}}{4\pi \varepsilon_{0}m}\left(\frac{1}{r_{ij}}+\frac{1}{\lambda_{D}}\right)\left(-\frac{x_{ij}}{r_{ij}^{2}}\right)e^{-\frac{r_{ij}}{\lambda_{D}}}dt. \end{array}$$

Similarly, the equations of velocity and position of node *i* at (n+1)dt in *y*-axis and *z*-axis can be written as

(13) $$\begin{array}{c} \left(1+\frac{\gamma dt}{2}\right)v_{(+1)y}=v_{y}-\left(\frac{\gamma }{2}v_{y}+\frac{2k}{m}y_{i}\right)dt+\sum_{i\neq j}^{N}\frac{Q^{2}}{4\pi \varepsilon_{0}m}\left(\frac{1}{r_{ij}}+\frac{1}{\lambda_{D}}\right)\left(-\frac{y_{ij}}{r_{ij}^{2}}\right)e^{-\frac{r_{ij}}{\lambda_{D}}}dt. \end{array}$$

(14) $$\begin{array}{c} \left(1+\frac{\gamma dt}{2}\right)v_{(+1)z}=v_{z}-\left(\frac{\gamma }{2}v_{z}+\frac{2k}{m}z_{i}\right)dt+\sum_{i\neq j}^{N}\frac{Q^{2}}{4\pi \varepsilon_{0}m}\left(\frac{1}{r_{ij}}+\frac{1}{\lambda_{D}}\right)\left(-\frac{z_{ij}}{r_{ij}^{2}}\right)e^{-\frac{r_{ij}}{\lambda_{D}}}dt. \end{array}$$

Therefore, we can theoretically calculate the node deployment of a WSN inspired by dusty plasma crystallization in real 3D space and determine the whole network topology in detail.

## 3. Performance Evaluation Methods

In a two-dimensional WSN, the performance of a deployment algorithm can be tested by the comparisons between the final network distribution and a hexagonal structure. However, this cannot be used in a three-dimensional distribution. In this paper, we will verify the simulation results by following performance evaluation methods.

### 3.1. Moving Distance

In practical applications, the UAVs usually have limited battery energy and cannot fly a very far distance. The most significant energy consumption in the actual deployment is the mobility. In such a case, the normalized moving distance traveled by the UAVs can reflect the energy consumption of the whole network system with respect to one 3D deployment scenario. Here we introduced the moving distance as one measurement of system deployment performance.

Set $S_{0}^{i}$ be the initial position of the sensor node *i* and its position during the whole deployment process are $S_{1}^{i},S_{2}^{i},S_{3}^{i},\ldots ,S_{t-1}^{i},S_{t}^{i}$. Therefore, the displacement distance of node *i* can be obtained as

(15) $$D_{i}=\sum_{j=1}^{t}d\left(S_{j-1}^{i},S_{j}^{i}\right)$$

where *D* is the Euclidean distance between the positions calculated from the adjacent time steps. In such a case, the average moving distance and corresponding variance of all nodes in a WSN are

(16) $$\bar{D}=\frac{1}{N}\sum_{i=1}^{N}D_{i}=\frac{1}{N}\sum_{i=1}^{N}\sum_{j=1}^{t}d\left(S_{j-1}^{i},S_{j}^{i}\right)$$

and

(17) $$\sigma^{2}=\frac{1}{N}\sum_{i=1}^{N}{\left(D_{i}-\bar{D}\right)}^{2}.$$

### 3.2. Spatial Distribution Diversion

Usually, a three-dimensional topology is very difficult to characterize. Here we compare the system performance of the sensor network deployment with the spherical accumulation of truncated octahedrons, which is inspired by the comparison of two-dimensional spatial sensor network topology with a hexagonal structure. This evaluation criterion can be used to estimate the integrity of our deployment by mathematically calculating the similarity of sensor network deployment results.

The topology evaluation of a 3D sensor network can be compared with one good 3D stacking structure, which is generally accepted in theoretical analysis or real applications, such as the spherical stacking composed of truncated octahedrons. The assessment of differences can be estimated based on the radial distribution function in molecular dynamics simulations. It is well known that the radial distribution function in 3D space, as the spatial distribution function (SDF), can be defined as the ratio of local number density to the mean density of the particle

(18) $$\Omega \left(x,y,z\right)=\frac{\rho (x,y,z)}{\bar{\rho }}.$$

Herein we define the SDF of our network topology as the ratio of the density of the spherical shell to the average density, as we are studying the regular spherical distribution.

(19) $$S\left(r\right)=\frac{\rho (r)}{\bar{\rho }}$$

where *r* is the radius from the center of the whole sensor network.

Therefore, the similarity between the network topology of our algorithm and the spherical stacking composed of truncated octahedrons can be evaluated according to the difference of radial distribution function, namely Spatial Distribution Diversion (SDD), as follows

(20) $$SDD=\delta \left(\Omega ,\Omega_{T}\right)=\frac{\int_{0}^{r_{T}}\left|\right|S_{\Omega }\left(r\right)-S_{\Omega_{T}}\left(r\right){\left|\right|}^{2}dr}{\int_{0}^{r_{T}}\left|\right|S_{\Omega_{T}}\left(r\right){\left|\right|}^{2}dr}$$

where Ω is the network topology to be analyzed, $\Omega_{T}$ is the topology of the standard spherical stack of truncated octahedrons, and $r_{T}$ is the radial distance distributed to its center (which is also the range compared with truncated octahedron stack). It can be seen that, when the SDD value is smaller, the formed sensor network is more similar with the truncated octahedron stack. In such a case, the SDD can be used to evaluate the quality of the network formed by the 3D deployment algorithm. When the SDD value is large, the network is poor. On the other hand, while the SDD value is small, we think the network is good, which has the potential to achieve high coverage and stable state.

### 3.3. System Coverage Rate

The system coverage rate, which represents the sensor networks’ monitoring performance to the detecting region, is the most significant criterion for evaluating sensor networks in practical applications. Higher system coverage indicates that, if the sensor network in the target region is more comprehensive, the less vital data will be lost, which allows scientists to obtain a better network for the target area.

**Figure 1:**
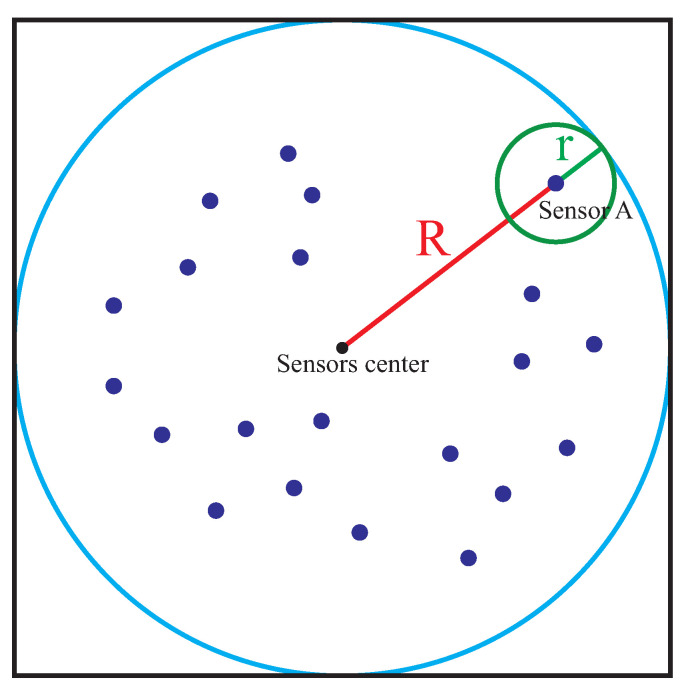
System Coverage Rate Schematic Diagram.

In order to evaluate the coverage effect of the final topology deployed by our 3D algorithm, we use the Monte Carlo algorithm to calculate the system coverage rate following by below steps, as shown in Figure 1.

(1) Set node *A* to be the sensor node farthest from the center of the sphere region when the network approaches the equilibrium state, and define the distance between the two nodes as *R*. If the sensing radius of a single node is $R_{S}$, the radius of the overall coverage area is $R_{a}=R+R_{S}$ and the corresponding entire coverage area should be $V_{a}=\left(4/3\right)\pi R_{a}^{3}$.

(2) Use the Monte Carlo algorithm to randomly select one sensor node and make the circumscribed cube of $V_{a}$ region. We take three random values X,Y,Z from the interval $\left[-R_{a},R_{a}\right]$ to obtain a three dimensional point (X,Y,Z). It is necessary to determine whether this point is within the coverage area by checking the condition $X^{2}+Y^{2}+Z^{2}<=R_{a}^{2}$. If this condition is satisfied, this test point will be chosen. Otherwise, we do the selection process again according to the above rules.

(3) Depending on the current position $\left(x_{i},y_{i},z_{i}\right)$ of node *i*, we mark the point within the range of a sensor if ${\left(x_{i}-X\right)}^{2}+{\left(y_{i}-Y\right)}^{2}+{\left(z_{i}-Z\right)}^{2}\leq r^{2}$.

(4) According to the number of test points, $p_{t}$, we can repeat procedures (2) and (3) for $p_{t}$ times and finally obtain the number of all marker points, which can be recorded as $p_{m}$. Therefore, the system coverage rate will be evaluated by $C=p_{m}/p_{t}$.

It should be noted that the Monte Carlo algorithm is only used to evaluate the system coverage rate. It is not a part of VFA-DP-3D algorithm to deploy the sensor node (with the GPS and UAVs). The Monte Carlo algorithm will help the users to check if the 3D network coverage rate is good enough or not at the beginning or any time step of the network deployment process, which can ensure that the final network can satisfy the user’s requirements.

### 3.4. System Utilization

In practical applications, when the coverage region increases, there will always be regions of recurrent detection, which wastes the hardware resources. The utilization is introduced here to evaluate the redundancy of sensor networks. Increasing system coverage while maintaining a high utilization rate is still very important.

Due to the uniqueness of the algorithm, we can observe that $R_{S}$, the key parameter determining coverage rate, does not participate in the calculation. It is obvious that the larger $R_{S}$ is, the larger the coverage value is, but at the same time, the area repeatedly covered by each node is also increasing. Therefore, utilization is used to evaluate the situation of repeated coverage between nodes.

(21) $$U=\frac{V_{a}C}{Nv}$$

where *v* represents the range that each node can cover, *n* represents the number of nodes, *C* represents coverage rate, and $V_{a}$ represents the maximum range covered by node distribution

(22) $$V_{a}=\frac{3}{4}\pi R_{a}^{3}$$

$R_{a}$ is defined in the same way as in the above subsection.

According to the Equation (21), when the whole 3D sensor network is deployed and reaches the equilibrium state, the change of the node sensing radius $R_{S}$ will dramatically change the size of *U* since $V_{a}$ and *v* are proportional to $R_{S}^{3}$. Therefore, by combining *C* and *U*, we can more comprehensively evaluate the quality of the distribution. We believe that a suitable $R_{S}$ may improve the network deployment, in which both *U* and *C* achieve a high value.

## 4. Simulation Analysis of 3D Network Deployment Experiments

Generally, one sensor node will include the given sensors and movement devices (such as GPS and UAV). It will have a corresponding sensing radius. Once the target region is determined, a deployment algorithm is needed to dynamically deploy all sensor nodes to the specified positions. Since we mainly concentrate on a large-scale WSN application, as for all simulation experiments in this paper, the number of sensor nodes was set to be N=3000 in the whole target space. Furthermore, the initial parameters were chosen based on the fundamental experiments by Ma and Bhattacharjee [30]. The typical settings are Q=15,700e, $m=5.2\times 10^{-13}$ kg, v=2.7 s${}^{-1}$, $k=2.4\times 10^{-13}$ kg/s${}^{2}$, $\lambda_{D}=0.0526$ μm, and $R_{S}=\sqrt{3}\lambda_{D}$. In most WSN applications, sensor communication and the corresponding routing protocol affect system performance, so they are very important topics in theoretical modeling and field tests. In this paper, we do not consider any communication and mechanical parts since our VFA-DP-3D is a theoretical node-deployment solution. However, in real applications, users can easily obtain the exact node positions based on the 3D coordinates in the target region to achieve the real node deployment.

**Figure 2:**
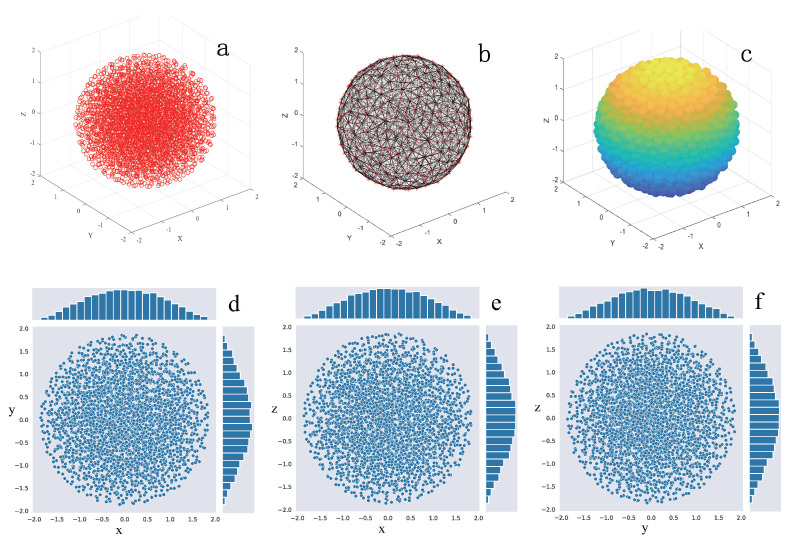
(**a**) 3D nodes diagram. (**b**) 3D topology. (**c**) 3D balls diagram. (**d**) The projection of the XoY plane. (**e**) The projection of the XoZ plane. (**f**) The projection of the YoZ plane.

Firstly, Figure 2a–c give one typical case result of the final network deployment structure for a 3D large scale WSN application, such as the 3D node diagram, the 3D network topology and the 3D ball diagram, respectively. We also present two dimensional projection of the whole 3D network on the XoY, XoZ and YoZ planes, as shown in Figure 2d–f. Generally, the overall network structure is spherical. It can be seen that, from the network topology and ball diagram, the distribution of the three dimensional spherical node network is relatively uniform. However, it still needs further evaluations for the detailed performance of the network deployment.

**Figure 3:**
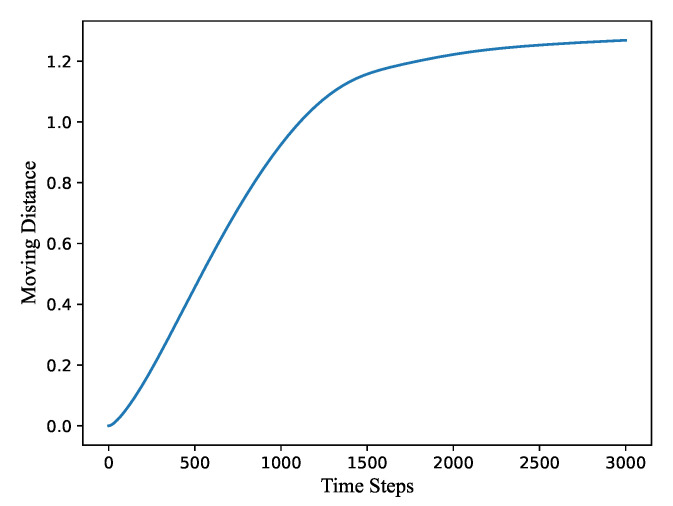
The variation of the total moving distance from all sensor nodes depending on the calculation time steps.

Figure 3 shows the variation of the total moving distance from all sensor nodes depending on the calculation time steps. During the 0 to 1000 time steps, the node moving distance increases very fast because all nodes are adjusting the network structure under the action of deployment algorithm. After 1500 time steps, it tends to stabilize and grow slowly. The network deployment has been roughly completed, after which the nodes can adjust positions gently to achieve a better network state and finally reach a convergence state around 2500–3000 steps.

**Figure 4:**
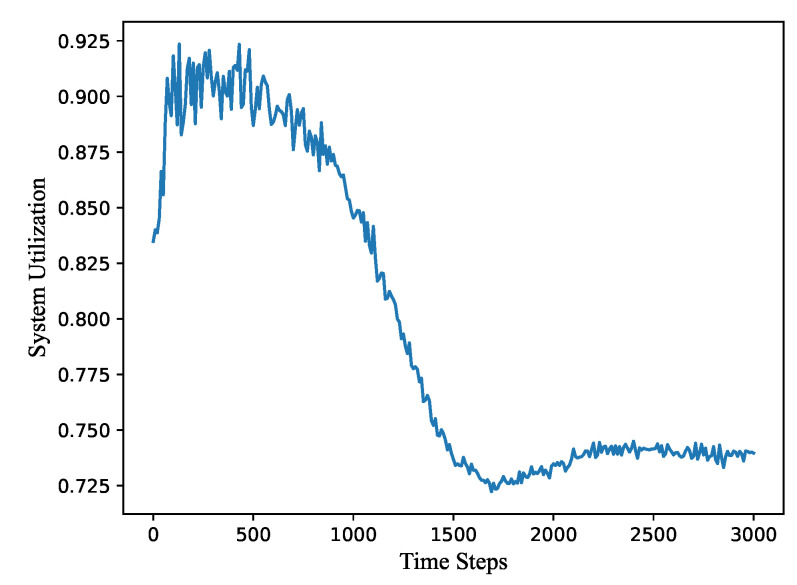
The variation of the system utilization depending on the calculation time steps.

Figure 4 gives the variation of the system utilization depending on the calculation time steps. It increases at the first 0–500 time steps. The reason is that, with the progress of the algorithm, all nodes become sparse first and a certain distance is maintained between all nodes. After 500 steps, the system utilization decreases. It can be inferred that the deployment of nodes is more regular and has a certain contraction trend, After 1500 steps, the network deployment tends to be stable, but there are still some small fluctuations. The whole 3D network reaches a convergence state after 2500–3000 steps and the utilization rate is about 0.73. It should be noted that the convergence of the system utilization is very consistent with that of the moving distance, around 1700 time steps, which can also confirm that these two performance evaluations are correct.

**Figure 5:**
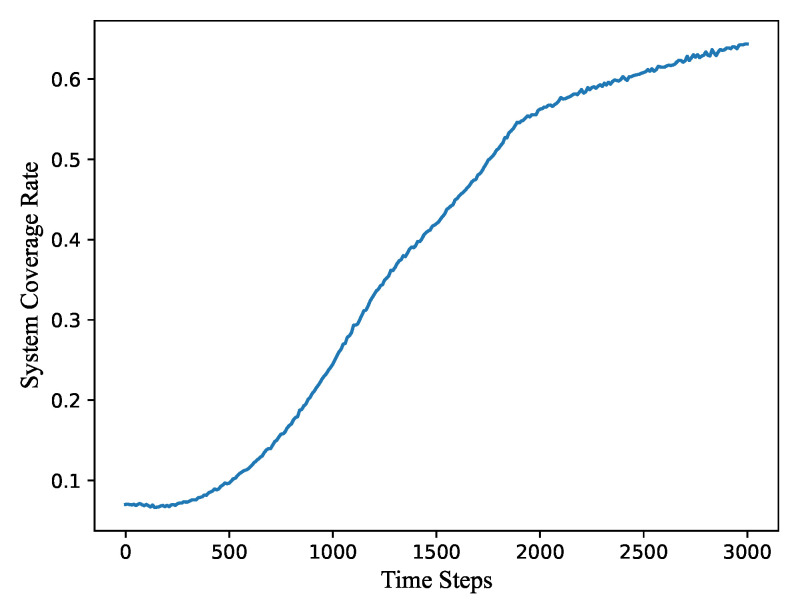
The variation of the system coverage rate as a function of the calculation time steps.

As for the system coverage rate, Figure 5 presents its variation as a function of the calculation time steps. It can be observed that the coverage rate was only 0.1 at the beginning. At 0–500 steps, the coverage rate slightly decreases and then increases. It means that the deployment range of nodes tends to expand and then shrink at the first 500 steps. After 500 steps, all nodes continue to shrink regularly. After 1700 steps, when the utilization rate and the moving distance begin to stabilize, the coverage rate begin to rise slowly. It suggests that our 3D algorithm still has a small-scale adjustment process to obtain a better network structure. Finally, the coverage rate reaches around 0.6 while the whole network has a high utilization.

In order to test the validation of the Monte Carlo algorithm on the 3D network coverage rate, Table 1 gives the final values of the coverage rate at the 3000th time step, for three independent running cases, based on different number of test points $p_{t}$. It can be seen that, under a different number of test points, the coverage rate does not change much for all three cases. Those very small differences are due to the difference of initial distribution and the calculation error of the Monte Carlo algorithm itself. We think that these small differences can be ignored and the Monte Carlo algorithm does present a good evaluation for the calculation of the system utilization.

**Figure 6:**
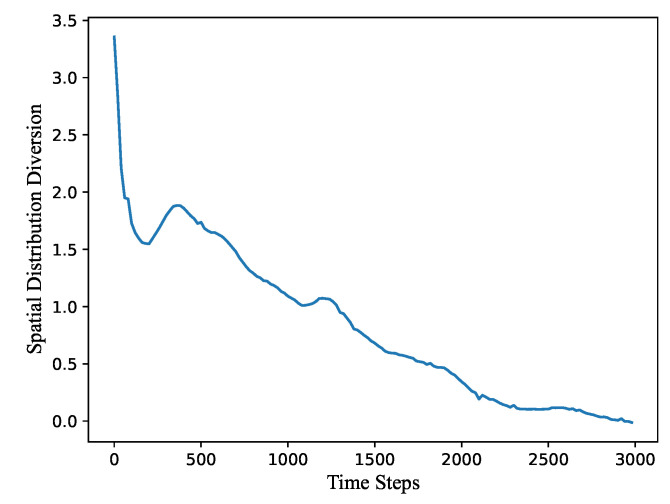
The variation of the spatial distribution diversion (SDD) value as a function of the calculation time steps.

Figure 6 shows the variation of the spatial distribution diversion (SDD) value as a function of the calculation time steps. We can observe that, when the nodes in the initial state are randomly distributed at the beginning, the network structure is very chaotic and the SDD value is large. During the first 200 time steps, the network quickly tends to be regular and the SDD value decreases sharply. During the 200–500 steps, all nodes have a process of reverse acceleration due to the action of the formula. According to the above discussions on the system utilization and the coverage rate, it also confirms that all nodes do tend to expand first and then shrink. After 500 steps, the SDD value stably decreases and the network topology becomes more close to the truncated octahedron stack based on our 3D deployment algorithm. Finally, the convergence state is obtained at 3000 steps and the corresponding SDD value tends to be 0.

## 5. Parameter Effect of the Debye Length $\lambda_{D}$ and Sensing Radius $R_{S}$ on the 3D Deployment

Based on the basic physics theory of dusty plasma [30], the Debye length $\lambda_{D}$ has an important impact on the operation of the algorithm, which dramatically determines the calculation scale of each algorithm. In this section, we further concentrate on the parameter effect of the Debye length $\lambda_{D}$ on the performance evaluations of our 3D WSN deployment algorithm.

As shown in Table 2, we conducted a large number of experiments to calculate the average moving distance and the variance of the moving distance under different values of $\lambda_{D}$. It can be seen that, with the continuous increase of $\lambda_{D}$, both the average moving distance and the corresponding variances decrease. Moreover, their fluctuation ranges have also been reduced. This significantly suggests that the increase of $\lambda_{D}$ will save the energy cost of the whole network.

**Figure 7:**
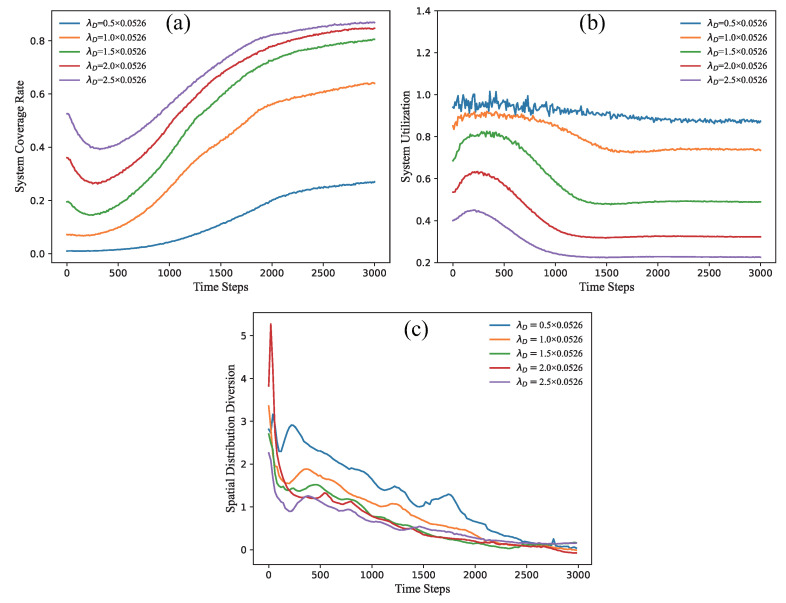
(**a**) The change of system coverage rate, (**b**) the change of system utilization rate, and (**c**) the variation of the spatial distribution diversion depending on different $\lambda_{D}$ as a function of the calculation time steps.

Figure 7 gives the variations of the coverage rate, the system utilization, and the spatial distribution diversion (SDD) respectively, depending on a different Debye length $\lambda_{D}$ as a function of the calculation time steps. Generally, the coverage rage will be larger when $\lambda_{D}$ increases, but the system utilization will decrease. It is very reasonable because the $\lambda_{D}$ has the shielding effect in dusty plasma theory, which will push all nodes outward the system center. In such a case, the system utilization correspondingly becomes worse. Similarly with Figure 6, the SDD values decrease when the time steps increase. The convergence speed of the SDD is fastest at the 0–200 steps and then slightly rises at 500–1000 steps, which is related to the reverse of velocity and acceleration in the formula. It can be roughly seen that, when the $\lambda_{D}$ is larger, the SDD will decrease faster at the early stage. After 2500 steps, the SDD values from different $\lambda_{D}$ tend to be stable and also converge to around 0, but the final SDD value of the distribution with larger $\lambda_{D}$ will also be larger. Based on these experiments, we can conclude that the increase of the Debye length $\lambda_{D}$ will not change the final distributions of 3D sensor network deployment. Our 3D algorithm can work well and perform a very good stack structure of the truncated octahedrons.

**Figure 8:**
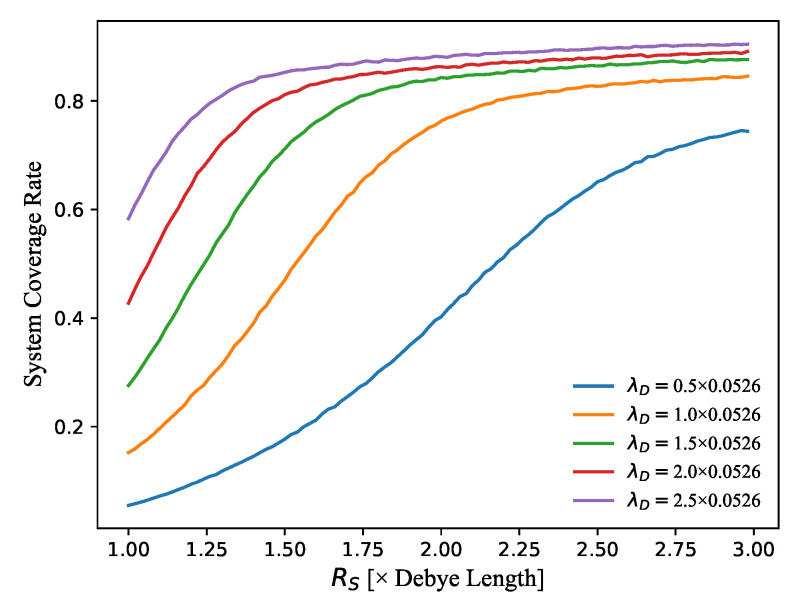
The changes of the final system coverage rate at 3000 time steps developed by our 3D algorithm as a function of node sensing radius $R_{S}$, corresponding to different values of $\lambda_{D}$.

On the other hand, it is still worth checking the influence of the node sensing radius $R_{S}$ on the performance of the 3D network deployment. Based on the formulae mentioned above, we have done a lot of experiments. Figure 8 presents the changes of the final system coverage rate at 3000 time steps developed by our 3D algorithm as a function of the node sensing radius $R_{S}$, corresponding to different values of $\lambda_{D}$. It can be clearly seen that, when the $\lambda_{D}$ value is fixed, the system coverage rate will be larger when $R_{S}$ increases. A large $R_{S}$ can increase the sensing range of a single node, which will finally improve the whole coverage area. Besides, the system coverage rate for a large $\lambda_{D}$ reaches a maximum value more quickly. This proves that, for a real WSN application, the $R_{S}$ cannot be increased randomly since it will dramatically use the system energy and need a higher hardware cost. Furthermore, it is also found that, when the $\lambda_{D}$ increases, the final coverage rate usually becomes higher.

**Figure 9:**
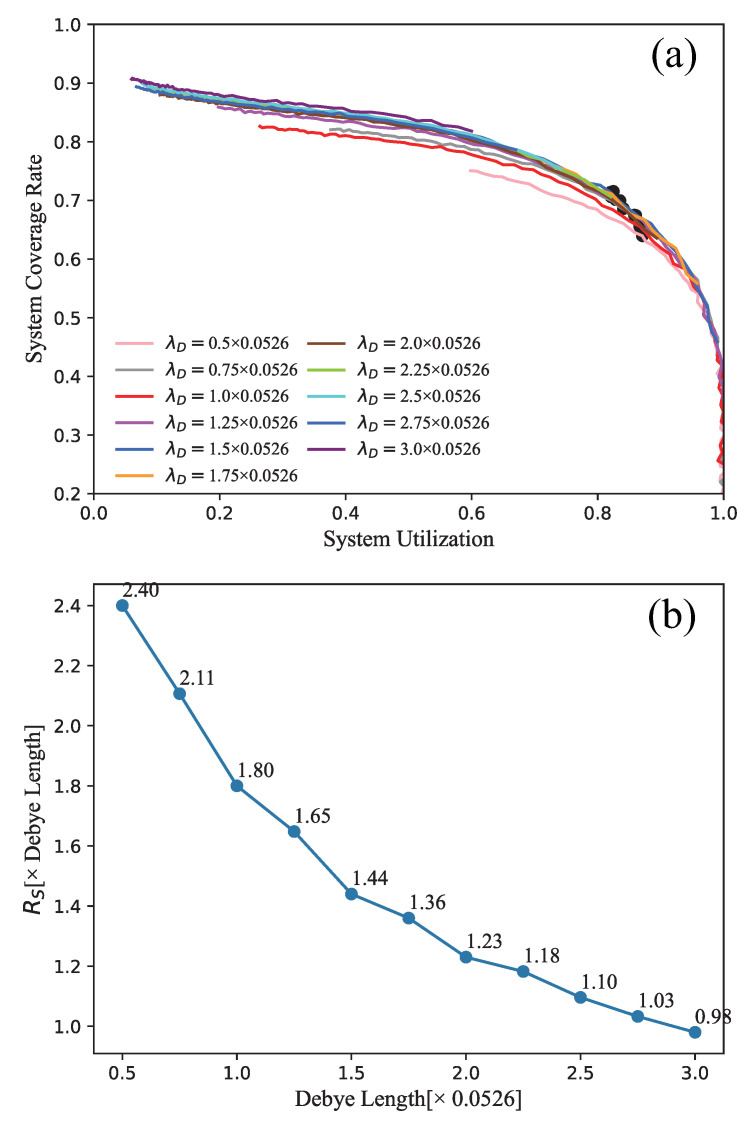
(**a**) The variation of coverage rate and the system utilization depending on different $\lambda_{D}$. (**b**) optimal $R_{S}$ for different $\lambda_{D}$.

Moreover, in order to select the best $R_{S}$ for better coverage rate and system utilization corresponding to a different Debye length $\lambda_{D}$, we compare the relationship between the final coverage rate *C* and system utilization *U* at 3000 time steps deployed by our 3D deployment algorithm. As shown in Figure 9a, those curves give the variations of the final coverage rates and system utilization for $\lambda_{D}$ = 0.5, 0.75, 1.0, 1.25, 1.5, 1.75, 2.0, 2.25, 2.5, 2.75, 3.0 × 0.0526. We can find that all coverage rates may reach a maximum value around 0.8, which is consistent with the above analysis. For different $\lambda_{D}$, when $\lambda_{D}$ is larger, *C* is generally larger and *U* is generally smaller. When $\lambda_{D}$ is smaller, *U* is generally larger and *C* will be smaller. Especially, for a fixed $\lambda_{D}$, when the network requires better system utilization, the coverage rate has to decrease. It can be generally seen that the ideal results show U∼0.8 and C∼0.7.

Furthermore, If we choose the maximum value of C×U marked by black dots in Figure 9a as the best system performance, the corresponding optimal $R_{S}$ can be determined by Equations (21) and (22) and is shown in Figure 9b. It gives the changes of the optimal $R_{S}$ depending on the Debye length $\lambda_{D}$. We can find that the optimal $R_{S}$ for a sensor node in a 3D WSN application decreases when the $\lambda_{D}$ increases. It shows that, when a small $\lambda_{D}$ is used as the parameter to calculate the network deployment, a relatively ideal system utilization *U* and coverage rate *C* can be obtained by using a larger $R_{S}$.

## 6. Conclusions

In this paper, for the first time, we present a three dimensional network deployment algorithm inspired by physical dusty plasma crystallization theory in large scale WSN applications. We summarize our main conclusions and contributions as follows:

(1) The 3D node deployment algorithm (VFA-DP-3D) and corresponding equations developed from the dusty plasma theory are presented. We also introduced four kinds of performance evaluation methods in 3D space, such as the moving distance, the spatial distribution diversion, system coverage rate, and the system utilization. The SDD parameter is compared with the truncated octahedron distribution to intuitively characterize the quality of the whole network. Based on the detailed analysis, our virtual force algorithm can work well in a 3D space for large scale WSN applications, which has good system coverage and small energy cost (moving distance). According to the SDD value, we observe that our algorithm can quickly deploy the scattered sensor nodes to a relatively regular state in the first 200 steps. However, due to the influence of the algorithm formulae, most of the nodes will have slight fluctuations because of the reverse distribution of acceleration during the 500–1000 steps. However, after 1500 steps, the node deployment tends to be stable and reaches a good convergence state.

(2) Since the Debye length is the most important parameter in a dusty plasma algorithm, we further discussed the parameter effect of $\lambda_{D}$ on the 3D deployment performance. A larger $\lambda_{D}$ corresponds to the wider final deployment range and the sparser distribution. It should also be noticed that, when $\lambda_{D}$ is larger, the SDD value has a faster convergence speed, which means that the whole network takes less time to reach a better deployment state. Besides, the corresponding moving distance becomes smaller and has less variance, which also indicates less system energy consumption. After $\lambda_{D}$ is fixed, the density state of the distribution needs to be further determined. We can then evaluate the whole system performances of the final network state, such as the coverage rate *C* and utilization *U*, by artificially varying $R_{S}$. When $\lambda_{D}$ is smaller, the energy consumption is larger and the convergence speed is slower, so that a larger $R_{S}$ is required to achieve the ideal coverage effect. When we look at the final performance of WSN deployment by combining the system coverage rate and the system utilization, it suggests that our algorithm can make WSN obtain a greater coverage rate at a certain point with a higher utilization. After finding the corresponding optimal sensing radius at that point, we can also use this value to adjust network settings in the practical applications.

In a summary, our 3D-VFA-DP algorithm can quickly complete an overall 3D network deployment and then dynamically adjust some parameters to achieve a better distribution. In our opinion, there are two ways to use our method in the practical applications: (1) If the users need to deploy a very large-scale sensor network in a real 3D application, our method can quickly calculate the exact positions for all nodes. All four evaluation methods can be combined to give the convergence and the whole performance of the calculated 3D UAV-assisted wireless sensor network before the actual deployment process. After obtaining the network topology and those positions, all UAVs can fly to their given positions to achieve the sensor network and collect information. In such a case, our method provides a possible virtual force algorithm derived from the physical dusty plasma theory, which can indeed form a good spherical stack of truncated octahedrons. (2) On the other hand, the users can randomly spread all sensor nodes (sensors carried by UAVs) in a target region at the beginning. Then, all UAVs will be driven by the virtual force under the effect of Yukawa potential to the calculated positions. In such a case, the sensor nodes may be autonomously deployed to a spherical stack of truncated octahedrons in a 3D space. The Yukawa potential manages the distance between nodes throughout the process. This is a self-deployment by all UAVs. Generally, users can terminate the algorithm calculation based on the application requirements and the real-time situation of four evaluation results to avoid wasting too much computing resources.

In the near future, a lot of WSN applications will require more and more sensor nodes for a large-scale node deployment. How to use a 3D deployment method still requires further experimentation. We understand that our paper mainly shows a virtual force algorithm in simulations, but it also gives some new insights to 3D WSN applications for the users and engineers. Furthermore, this algorithm may be combined with other self-deployment methods to improve their location accuracy and achieve a hybrid optimization. It can be significantly used in some 3D WSN applications.

## Figures and Tables

**Table 1:** Coverage rates for different number of test points in Monte Carlo calculation.

Different Number of Points	Case 1	Case 2	Case 3
$p_{t}$ = 1 $\times 10^{4}$	0.6134	0.6106	0.6056
$p_{t}$ = 1 $\times 10^{5}$	0.6157	0.6097	0.6086
$p_{t}$ = 2 $\times 10^{5}$	0.6162	0.6129	0.6078
$p_{t}$ = 5 $\times 10^{5}$	0.6140	0.6103	0.6063

**Table 2:** The ranges of average moving distances and their variances corresponding to different $\lambda_{D}$ values.

Different $\lambda_{D}$	Average Moving Distance	Variance of Moving Distance
$\lambda_{D}$ = 0.5 × 0.0526	1.4653∼1.5113	0.2292∼0.2314
$\lambda_{D}$ = 1.0 × 0.0526	1.2542∼1.2815	0.1578∼0.1654
$\lambda_{D}$ = 1.5 × 0.0526	1.1204∼1.1341	0.1003∼0.1107
$\lambda_{D}$ = 2.0 × 0.0526	1.0201∼1.0317	0.0807∼0.0824
$\lambda_{D}$ = 2.5 × 0.0526	0.9207∼0.9295	0.0562∼0.0573

## Data Availability

Not Applicable.
